# An open-type microdevice to improve the quality of fluorescence labeling for axonal transport analysis in neurons

**DOI:** 10.1063/1.5090968

**Published:** 2019-05-09

**Authors:** S. Yokoyama, A. Otomo, S. Hadano, H. Kimura

**Affiliations:** 1 Micro/Nano Technology Center, Tokai University, Hiratsuka, Kanagawa 259-1292, Japan; 2 Department of Mechanical Engineering, School of Engineering, Osaka Institute of Technology, Kita-ku, Osaka 530-8568, Japan; 3 Department of Molecular Life Sciences, Tokai University School of Medicine, Isehara, Kanagawa 259-1193, Japan; 4 Department of Mechanical Engineering, School of Engineering, Tokai University, Hiratsuka, Kanagawa 259-1292, Japan

## Abstract

Abnormal axonal transport of vesicles as well as organelles in a particular set of neurons is implicated in the pathogenesis of many neurodegenerative diseases such as amyotrophic lateral sclerosis, Alzheimer's disease, and Parkinson's disease. Although various types of microfluidic multicompartmental devices with closed microchannels have been recently developed and widely used for axonal transport analysis, most of the existing devices are troublesome and time-consuming to handle, such as culture maintenances, sample collections, and immunocytochemistry. In this study, we overcome such inherent shortcomings by developing a novel open-type device that enables easy cell maintenance and sample collections. In our device, microgrooves instead of microchannels were directly fabricated on a glass substrate, thereby making possible a high-resolution optical observation. Compared with the conventional closed-type devices, our newly designed device allowed us to efficiently and precisely label the axonal acidic vesicles by fluorescent dyes, facilitating a high-throughput analysis of axonal vesicular transport. The present novel device, as a user-friendly and powerful tool, can be implemented in molecular and cellular pathogenesis studies on neurological diseases.

## I. INTRODUCTION

Each neuron consists of a nucleus and a cell body, often referred to as a soma, from which multiple dendrites extend like branches. A single axon, typically longer than the dendrites, extends and innervates to other neurons or cells elsewhere in the body. Axons act as an interneuron cable that is tasked with information transmission.^1,2^ Several neurological diseases, such as amyotrophic lateral sclerosis (ALS), Alzheimer's disease (AD), and Parkinson's disease (PD), have been known to be caused by nerve axon dysfunction.

ALS belongs to a heterogeneous group of progressive neurodegenerative disorders characterized by the selective loss of both upper motor neurons in the cerebral cortex and lower motor neurons in the brainstem and spinal cord.^3,4^ This disease frequently results in respiratory failure within 3–5 years. Although ALS has been extensively studied and is a well-known form of motor neuron diseases, its molecular pathogenesis has not fully been understood yet.

Recent works^5–8^ have suggested that abnormal proteolytic pathways mediated by autophagy in neurons are involved in the pathogenesis and progression of ALS. Autophagy is one of the key mechanisms by which damaged proteins and organelles are removed and degraded. During this process, unnecessary or dysfunctional proteins are isolated from the rest of the neurons and engulfed within a double-membraned vesicle known as an autophagosome. Autophagosomes are transported via axonal transport and fused with lysosomes; the contents including these proteins are then degraded and recycled.

Hadano *et al.* have discovered that the saccule membrane containing granular/osmiophilic aggregates and autophagosome-like vesicles produced by autophagy in the spinal cord of an ALS mouse model was accumulated with progress of disease.^9,10^ Similar pathological phenomena have also been detected in human ALS patients. However, it has not been proved that these pathological phenomena arise from transport disorders of autophagosomes in the axons.

The axonal transport of particular vesicles and organelles has been analyzed in neurons on culture dishes. However, because of difficulties in controlling the growth direction of axons and to distinguish between axons and dendrites of neurons on cell culture dishes, it is almost impossible to perform quantitative analysis of vesicles and/or organelle transport in the axon.

Taylor *et al.*^11,12^ developed a microfluidic multicompartmental device for neuroscience research. Their device has microgrooves that connect two compartments and facilitate controlled growth and isolation of the axons from their cell bodies. Through their device, they were able to distinguish between the cell bodies and axons and quantitatively evaluate axonal transport. Similar devices have been developed by many workers^13–19^ to address a broad range of topics within the realm of ALS disease research. In this type of multicompartmental device, sample preparations are time-consuming, and sophisticated skills are required to conduct the desired experiments. Although microfluidic multicompartmental devices have been widely applied, none of those with a closed-microchannel design can be easily employed to collect samples or perform culture maintenance or immunohistochemical analysis. Moreover, since a large number of experiments are typically needed to quantitatively evaluate axonal transport, the conventional devices are not suitable for such experiments.

Subsequently, Park *et al.*^20^ developed an open-type device to overcome the evident limitations of conventional axon growth research in immunohistochemical analysis. They demonstrated that maintenance and sample collections could be easily and efficiently performed using their device. Since the bottom surface of this device is, however, fabricated with polydimethylsiloxane (PDMS), a high-resolution optical observation is not feasible. This means that three-dimensional structural observation using a confocal microscope is impossible. In an effort to circumvent such limitations, we propose an optically observable open-type device with superior usability using reactive ion etching (RIE) to etch microgrooves into a cover glass. The microgroove structures are etched into the substrate in the open-type device, and hence the process of cell manipulation is not so complicated as that for closed-type devices. We report here the functional evaluation of the proposed device for the high-throughput analysis of neurons axonal transport.

## II. MATERIALS AND METHODS

### A. Design of microdevice

**FIG. 1.**
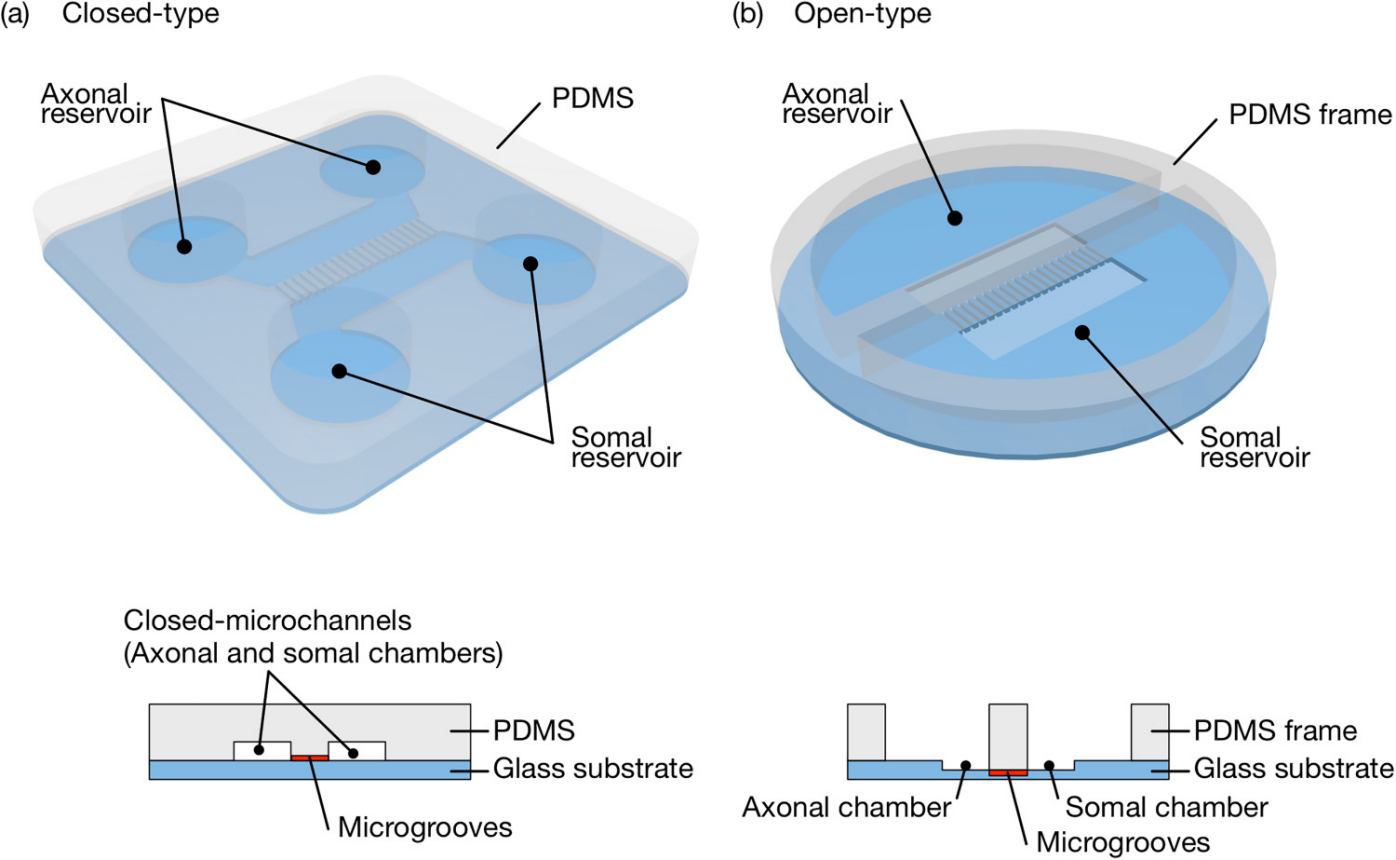
Overall and cross-sectional schematics of a conventional closed-type device (a) and the proposed open-type device (b). In both devices, the somal reservoir with cell bodies and the axonal reservoir with extending axons are separated by microgrooves.

The conventional microfluidic multicompartmental device for neuroscience research proposed by Taylor *et al*. consisted of two separate compartments connected by microgrooves.^8^ These two compartments were connected to two ports with closed microchannels using the hydrostatic pressure difference between the two compartments to prevent water immersion via resistance. They have the ability to contain and isolate a biomolecular insult, but their closed-microchannel design prevents their device from being easily applied for culture maintenances, sample collections, or immunohistochemical analysis [Fig. 1(a)]. To solve this and other related problems, we have developed an optically observable open-type device for simple cell maintenances and easy sample collections. The proposed device has two reservoirs separated by microgrooves without top shields. The base of the microgrooves of the device proposed here is on a glass substrate instead of a PDMS substrate, which is also suitable for high-resolution optical observation [Fig. 1(b)].

### B. Fabrication

**FIG. 2.**
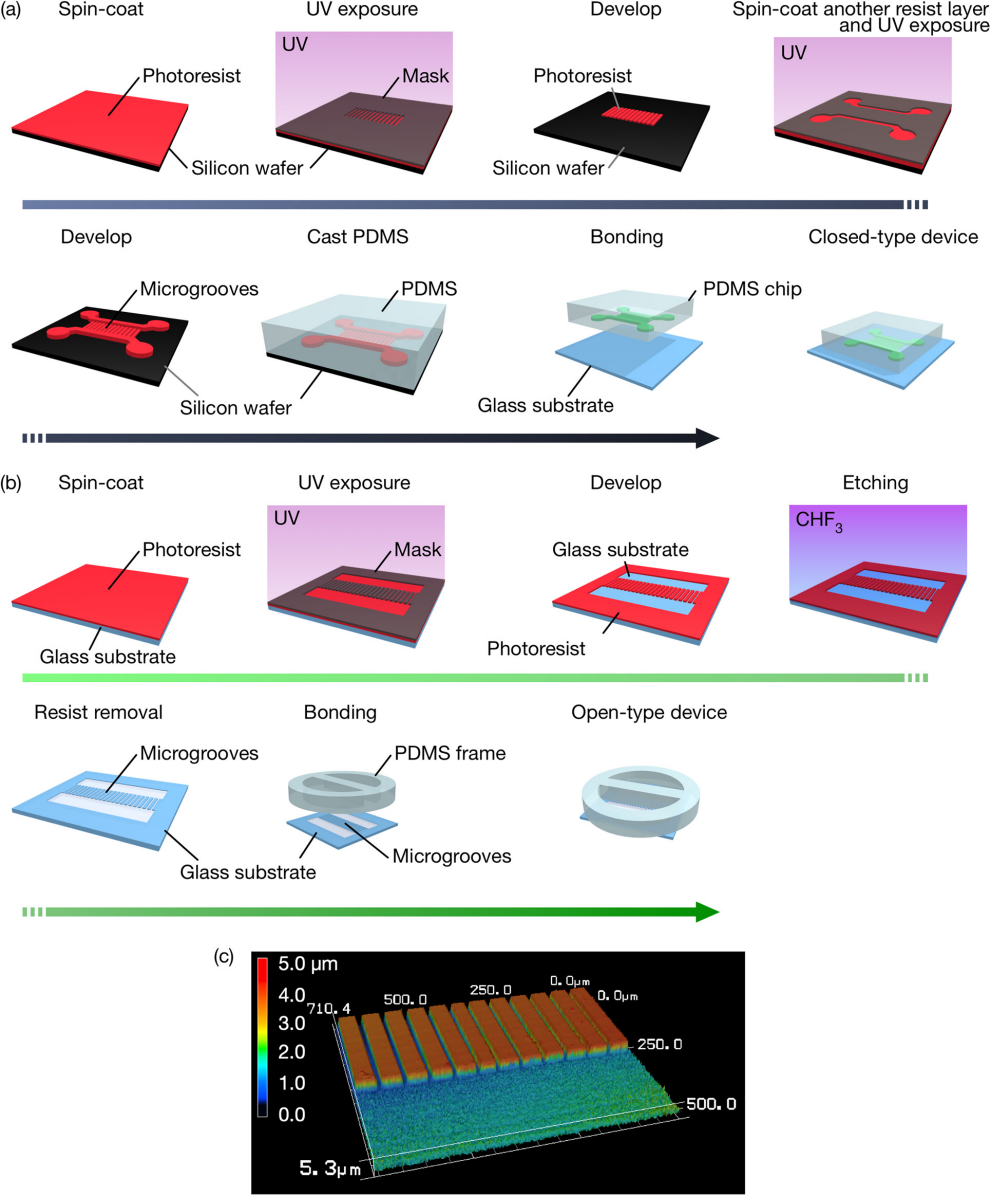
Schematic of the fabrication process for the closed-type device (a) and the open-type device proposed (b), and a laser microscope image of the microgrooves fabricated on a cover glass (c).

Both closed-type and open-type devices were fabricated and used for the functional evaluation in experiments. The closed-type device was fabricated in PDMS using soft lithography technique as shown in Fig. 2(a). A mold master was made by patterning two layers of a negative-type photoresist (SU-8, MicroChem). The first layer was used to construct the microgrooves. The size of the grooves was designed to limit the neurons in the somal chamber while allowing the growing nerve axons to cross from one chamber to another. The size of the microgroove is 1000 *μ*m in length, 4.5 *μ*m in height, and 10 *μ*m in width. The 10 *μ*m width of microgrooves is sufficiently narrow so as to prevent the migration of approximately 20 *μ*m diameter neurons into the microgrooves. The second layer was used to create the chamber areas and consisted of two separate compartments connected by the microgrooves. A prepolymer of PDMS (Silpot 184, Dow Corning Toray) was mixed with a curing reagent at a 10:1 mass ratio and poured over the mold masters. Then, the PDMS was cured at 75 °C for 2 h followed by peeling of the solidified PDMS from the masters. The PDMS chip and a glass plate were then assembled using the plasma bonding method.

In the open-type device, the microgrooves were fabricated on a glass substrate with an RIE apparatus, and the size of each microgroove is identical to that of the closed-type device [Fig. 2(b)]. The PDMS frame was bonded to the glass substrate by using plasma bonding in order to separate the two reservoirs through the microgroove; this design ensures that a sufficient volume of medium is supplied to the axonal and somal compartments. Both photolithography and RIE were used to fabricate the proposed device. The former was implemented to fabricate a microgroove-patterned photoresist that could act as a protective layer during the RIE process. A positive-type S1813G photoresist (Rohm and Haas Electronic Materials) was spin-coated onto a cover slip at 490 rpm and solidified after baking at 115 °C for 60 s on a hot plate. Following open-air cooling, the photoresist on the cover slip was covered with a photomask in the desired shape of the microgrooves and then exposed to UV light at 45 mJ/cm^2^ for 8 s. Following photomask application, the photoresist was developed by employing an AZ-developer (AZ Electronic Materials) for 40 s and postbaked at 200 °C for 90 s. RIE was applied to the glass substrate under the conditions of a reactive CHF_3_ gas, a 20-sccm gas flow rate, 250 W RF power, and 2.0 Pa over a period of 155 min in an RIE apparatus (RIE-10NR, Samco). Thereafter, the photoresist remaining on the cover slip was removed by applying acetone solution. The structure of the resulting microgrooves, which was imaged via a shape measurement laser microscope (VK-X200, KYENCE), is shown in Fig. 2(c). The PDMS frame was bonded to the glass substrate in order to separate the somal and axonal chambers.

### C. Diffusion rate evaluation of chemical substances

Texas Red-labeled dextran was utilized as a fluorescence indicator to visualize the diffusion rate because its molecular size is comparable to that of a neurotrophic factor. The labeled dextran was, respectively, added to the somal reservoir of the open- and closed-type devices at a final concentration of 20 *μ*M and subsequently observed every 10 min using an inverted microscope (ECLIPSE Ti and DS-Ri2, Nikon). The water head between each reservoir was maintained at the same level to ensure measurement differences between the open- and closed-type devices. Since the diffusion rate depends only on the structure of each device, it is possible to quantitatively evaluate the diffusion rate of chemical substance between the devices.

### D. Preparation of primary neurons

Primary cortical neurons were isolated from mice embryos at embryonic day 14; details of the isolation method have been described in a previous report.^21^ Cerebral cortex tissue samples were dissected, removed, and immediately placed into 500 *μ*l of ice-cold Hank's Balanced Salts (HBSS) (Sigma). The supernatant of HBSS was removed using a small desktop centrifuge (Millipore). 0.25% trypsin-EDTA was added to incubate the samples at 37 °C for 10 min. Following removal of trypsin-EDTA, the tissue samples were treated with DNase (final 50 mg/ml) that was incorporated into the HBSS. After employing centrifugation to remove the DNase in the HBSS, 500 *μ*l of HBSS containing 20% (v/v) fetal bovine serum (FBS) was added. A pipette was used to dissociate the tissue pellets from the HBSS containing 20% FBS, and the dissociated samples were then centrifuged at 400 rcf for 5 min using a centrifuge (Centrifuge 5418, Eppendorf). This process was repeated until the cells had been sufficiently dissociated. All animal experimental procedures were approved by The Institutional Animal Care and Use Committee at Tokai University.

### E. Live-cell labeling efficiency evaluation

The live-cell labeling efficiency evaluation was conducted by staining tests using LysoTracker, which is an acidotropic dye that stains cellular acidic compartments, including lysosomes and autolysosomes. After employing the trypan blue assay to count the number of living cells, we introduced 10 *μ*l of the cell suspension, which had a cell number that had been adjusted to 2.5 × 10^5^, to a somal compartment using a micropipette. The device was leveled for 15–30 min until the cells had adhered to the substrate. Then, 250 *μ*l of neurobasal medium (GIBCO, Invitrogen) containing 2% (v/v) B-27 supplement (Invitrogen) was added to each axonal and somal reservoir, and then cultured at 37 °C. Twenty-four hours after cells were seeded in the device, a brain-derived neurotrophic factor (BDNF) was added to the axonal compartment at 1 ng/ml with B-27 supplement, to control the growth direction of the nerve axons. The medium was replaced every 24 h. The device was placed in an incubator, where the cells were cultured in standard culture conditions (5% CO_2_, 37 °C) for up to 8 days. For cell staining, 50 *μ*l of 50 nM LysoTracker solution was introduced into only somal reservoirs on each device. Then, fluorescence images were observed and captured for 60 min. Fluorescence intensities of the images were evaluated using ImageJ (NIH).

### F. Statistical analysis

All values are expressed as mean ± SD from at least triplicate experiments. Student's t test for paired and unpaired comparison, as appropriate, was performed and differences were considered to be significant when p < 0.05.

## III. RESULTS AND DISCUSSION

### A. Diffusion rate in fluorescent material administration experiments

Using a fluorescent material as a model of fluorescent staining dyes, we measured the diffusion rate in dye administration experiments in order to compare the performance of the proposed device with that of conventional devices. As previously mentioned, Texas Red-labeled dextran (MW 10 000) was adopted. In this experiment, we investigated whether the dextran molecules penetrated into the end of the microgrooves immediately after they were introduced into the reservoir.

**FIG. 3.**
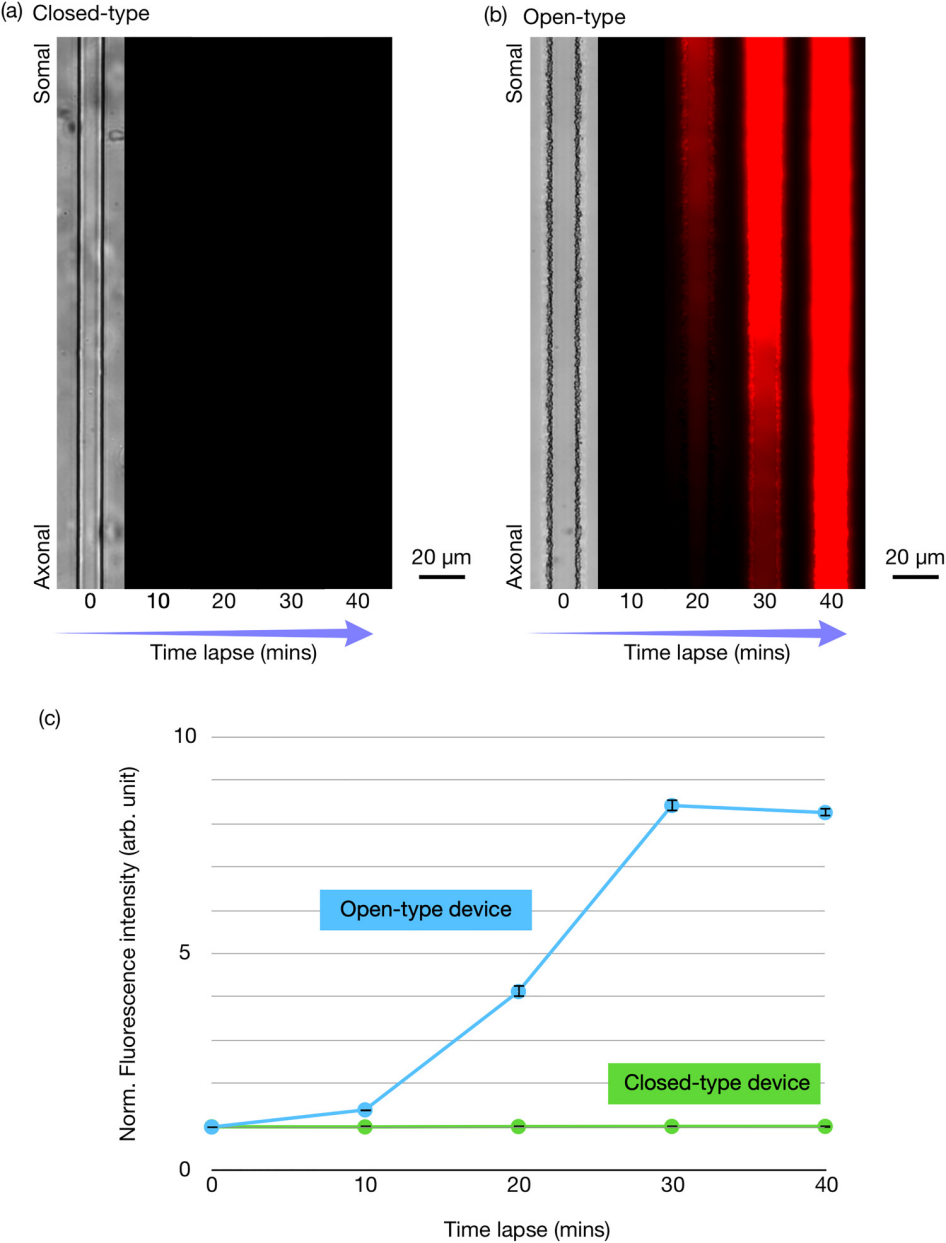
Dextran molecule penetration into the end of the microgrooves immediately after the dextran was introduced into the reservoir for the conventional closed-type device (a) and the proposed open-type device (b). Fluorescence intensity in the microgrooves as measured in intervals of 10 min (c).

Figure 3 illustrates the dextran diffusion rate results for the open- and closed-type devices. Figures 3(a) and 3(b) show the bright-field and fluorescent imaging results captured in 10-min intervals. In the proposed open-type device, the dextran molecules arrived at the axonal reservoir through the microgrooves. Figure 3(b) implies that, since the somal reservoir is directly connected to the microgrooves, our novel open-type device can be used to perform rapid fluorescent staining dye administration experiments. In contrast, Fig. 3(a) shows that the closed-type design of the conventional device did not allow a sufficient amount of dextran molecules to reach an area near the microgrooves, even after 40 min. We believe that a relatively long period of time is required for the drug to migrate to an area near the microgrooves, because the fluorescent material is introduced from the somal reservoir through the microchannels, and then moves toward the microgrooves.

Fluorescence intensities in the region of interest (ROI) were determined using ImageJ software (NIH). The ROI was defined as a region of 10 × 10 *μ*m^2^ at the center of the microgrooves. Figure 2(c) illustrates how the normalized fluorescence intensity in the microgrooves changed every 10 min. The fluorescence intensity in the open-type device is very clearly higher than that of the conventional closed-type device. The fluorescence intensity could not be further detected in the axonal reservoir of the conventional closed-type device. Conversely, the signal was detected immediately in the open-type device after the dextran molecules were introduced to the somal reservoir, and the dextran intensity reached its maximum value after 30 min. These results demonstrate that the open-type device is suitable for rapid fluorescent staining experiments.

Although a slow diffusion rate may be preferred to attract nerve axons, a fast diffusion rate would be preferable for the process of dyeing vesicles in axons in the microgrooves. In either case, since the open-type device does not incorporate the closed-microchannel design, we believe that the diffusion rate can be more easily changed.

### B. Comparison of efficiency of labeling

**FIG. 4.**
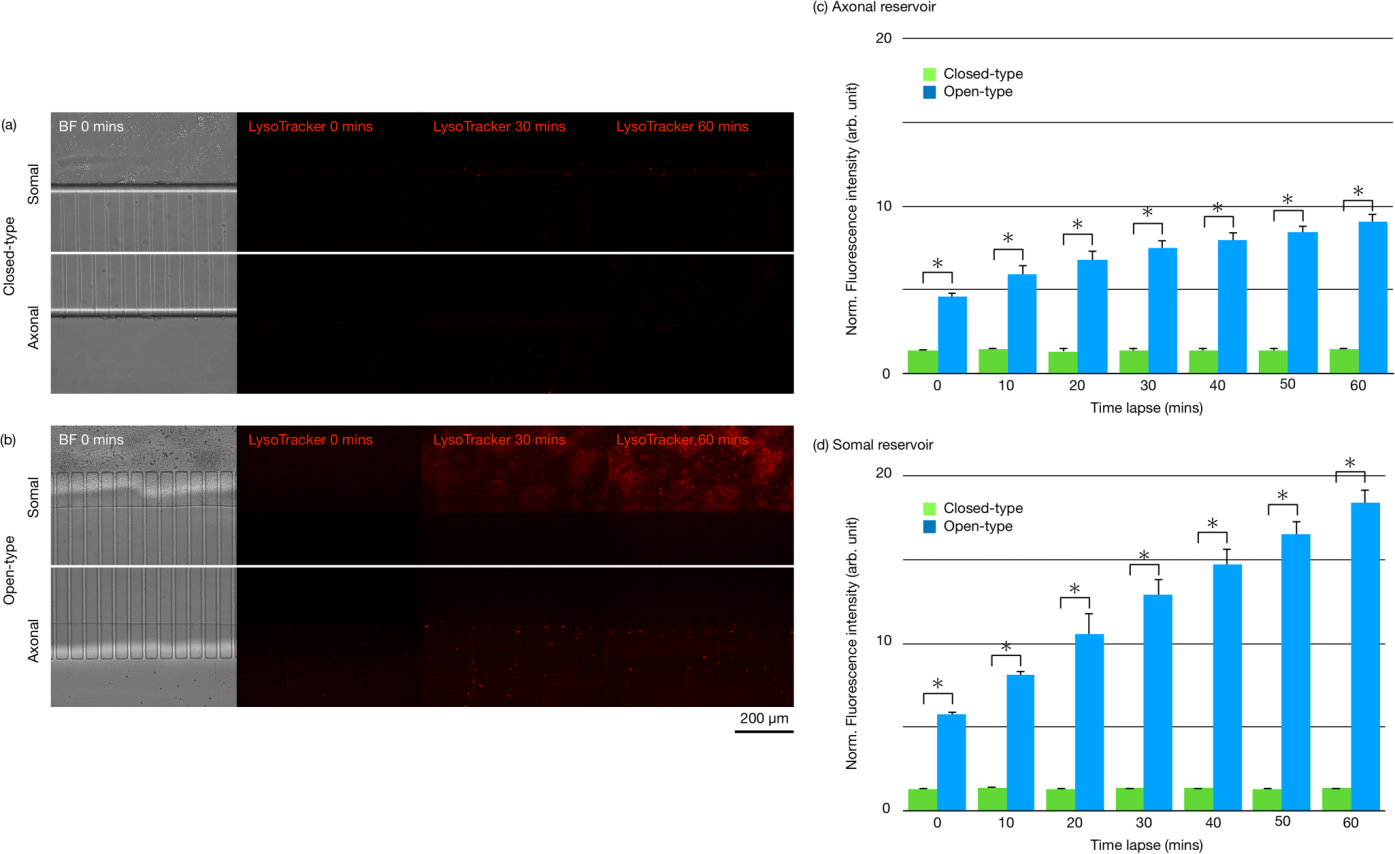
Fluorescence imaging results for the closed-type device (a) and the open-type device (b). LysoTracker, which visualizes the acidic vesicles in axons that contain autophagosomes, was applied into the somal reservoirs at a final concentration of 50 nM on cultured neurons. Fluorescence intensities of the axonal and somal reservoirs as measured and normalized in 10-min intervals at the axonal reservoir (c) and the somal reservoir (d). Data were normalized with a closed-type intensity level (*p < 0.05).

The labeling efficiency of the open-type device was more accurately evaluated with respect to the normalized fluorescence intensity and was quantified using the LysoTracker under the same conditions as those described in Sec. II D. Figures 4(a) and 4(b) show the fluorescence imaging results for the closed-type and open-type devices. The bottom glass layer of the devices allowed DIC microscopy. The fluorescence intensities of the somal and axonal reservoirs were measured and normalized in 10-min intervals [Figs. 4(c) and 4(d)]. The normalized fluorescence intensities of the open-type device axonal and somal reservoirs were significantly higher than those of the closed-type device. We also confirmed that acidic vesicles can be more efficiently labeled using the open-type device. Furthermore, the fluorescence intensity measured in the open-type devices was observed to increase over time. This suggests that more efficient labeling can be achieved with the proposed open-type device, as extremely shorter the exposure time to fluorescent probes is required than that of the open-type device.

### C. Axonal transport visualization

**FIG. 5.**
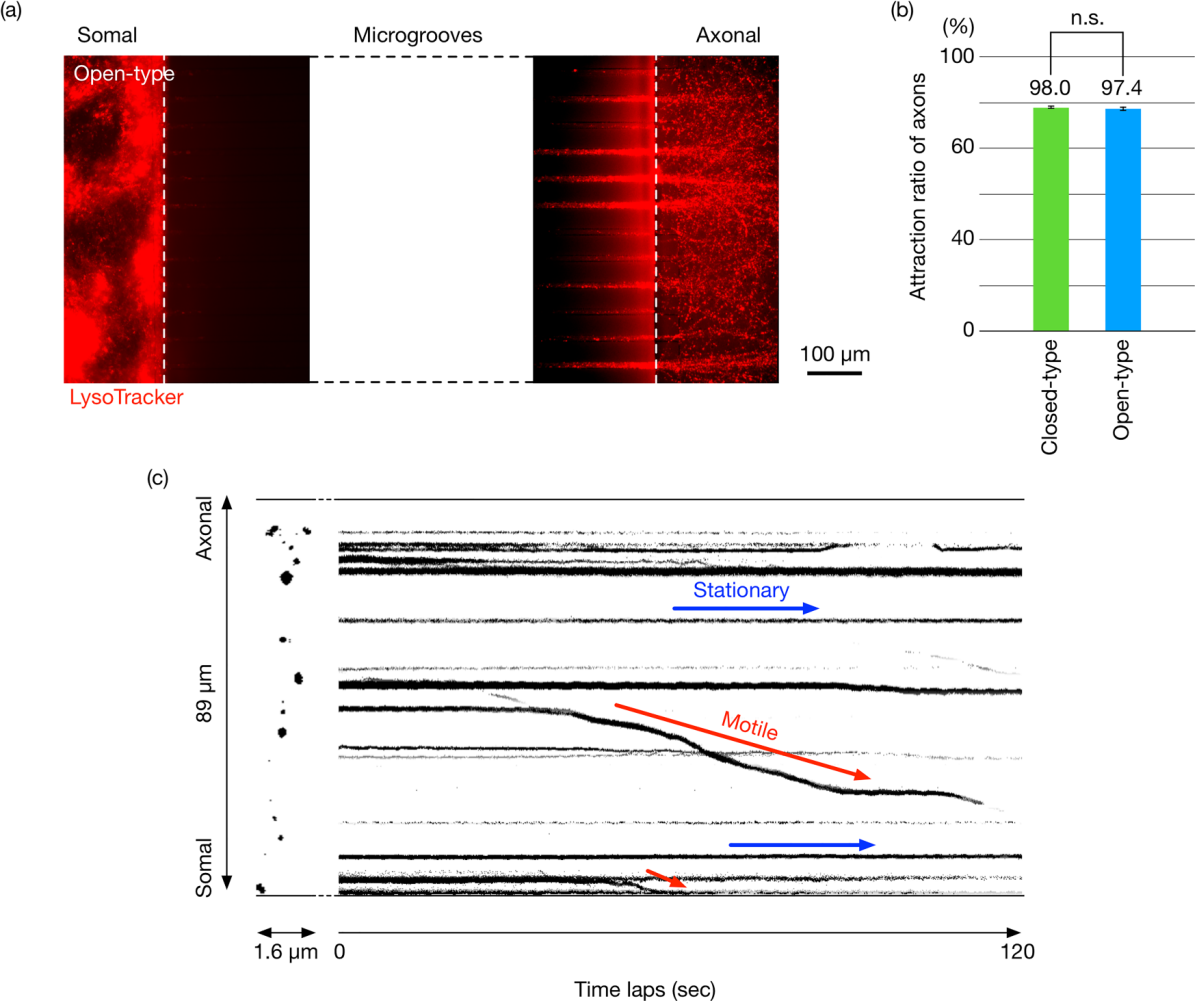
Acidic vesicles, including autophagosomes, in the somal and axonal reservoirs as visualized by LysoTracker staining as a probe in the proposed open-type device (a). Axons attraction ratio of the closed-type device and the open-type device (b). Time-laps images of acidic vesicle dynamics in neuronal cells. “Stationary” and “Motile” denote the stationary and moving vesicles, respectively (c).

We performed experiments to confirm that the proposed device is highly suited for the determination of the ratio between stationary and motile acidic vesicles, and the direction of moving vesicles in axons. Figure 5(a) shows the acidic vesicles, including the autophagosomes in the somal and axonal reservoirs, that were visualized by implementing LysoTracker as a probe in the proposed open-type device. Real-time axonal transport phenomena in the device were also observed with an all-in-one fluorescence microscope (BZ-9000, KEYENCE) and a stage-top incubator (INU-KI-F 1, TOKAI HIT) at 37 °C 5% CO_2_ (see Movie S1 in the supplementary material). We concerned that a fast diffusion rate in microgrooves may not be preferred to attract nerve axons. The attraction rate was calculated by dividing the number of microgrooves where one or more nerve axons are attracted by the total number of microgrooves. However, there was no significant difference in the axon attraction ratio between the closed-type device and the open-type device [Fig. 5(b)]. The results imply that the proposed device enables observation of the direction of information transmission in the axon passing through the microgrooves. Figure 5(c) indicates the trajectories of axonal transport of the acidic vesicles including autophagosomes in neurons. This result confirmed that the novel device enables us to evaluate the axonal transport quantitatively using a kymograph like the conventional devices. It is required to observe numerous nerve axons in attempts to efficiently and quantitatively grasp the axonal transport phenomena. We compared the axon numbers in the microgrooves counted by the novel and conventional devices. Consequently, we confirmed that a similar tendency for fewer cells was found in the vesicles moving in the axons.

## IV. CONCLUSIONS

We have successfully developed a novel device that can be employed as a user-friendly and powerful tool in the field of neuroscience. The device not only allows the dynamics of axonal vesicles in living cells to be analyzed but also improves the labeling efficiency. In the future, we are planning to use the proposed open-type device to conduct a quantitative evaluation of the axonal transport and use qPCR to perform RNA analysis in ALS mouse models.

## SUPPLEMENTARY MATERIAL

See the supplementary material for Movie S1 of acidic vesicle dynamics in neuronal cells visualized by LysoTracker playing at 10× speed.
